# Nurse-led intervention in the management of patients with cardiovascular diseases: a brief literature review

**DOI:** 10.1186/s12912-023-01422-6

**Published:** 2024-01-02

**Authors:** Xiaoqin Qiu

**Affiliations:** Department of Nursing, Guangxi Hospital Division of the First Affiliated Hospital, Sun Yat-Sen University, Qingxiu, Nanning, Guangxi 530022 P.R. China

**Keywords:** Nurse-led interventions, Nurse-led clinics, Coronary artery diseases, Cardiovascular risk factors, Percutaneous coronary intervention, Coronary artery bypass surgery, Nurse-led telemedicine

## Abstract

Coronary artery disease (CAD) is one among the major causes of mortality in patients all around the globe. It has been reported by the World Health Organization (WHO) that approximately 80% of cardiovascular diseases could be prevented through lifestyle modifications. Management of CAD involves the prevention and control of cardiovascular risk factors, invasive and non-invasive treatments including coronary revascularizations, adherence to proper medications and regular outpatient follow-ups. Nurse-led clinics were intended to mainly provide supportive, educational, preventive measures and psychological support to the patients, which were completely different from therapeutic clinics. Our review focuses on the involvement and implication of nurses in the primary and secondary prevention and management of cardiovascular diseases. Nurses have a vital role in Interventional cardiology. They also have major roles during the management of cardiac complications including congestive heart failure, atrial fibrillation and heart transplantation. Today, the implementation of a nurse-led tele-consultation strategy is also gaining positive views. Therefore, a nurse-led intervention for the management of patients with cardiovascular diseases should be implemented in clinical practice. Based on advances in therapy, more research should be carried out to further investigate the effect of nurse-led clinics during the long-term treatment and management of patients with cardiovascular diseases.

## Introduction

Today, cardiovascular disease (CVD), more specifically coronary artery disease (CAD) is on the rise [1]. Despite a decrease in the number of deaths, CAD is still one among the major causes of mortality in patients all around the globe [2]. Male patients, with an average age above 50 years and those with cardiovascular risk factors including a family history of CAD are at a higher risk of developing the disease [3]. It has been reported by the World Health Organization (WHO) that approximately 80% of cardiovascular diseases could be prevented through lifestyle modifications including healthy diet, control of blood sugar, sufficient amount of physical activities and permanent abstinence to smoking [4]. Those health promoting behaviors have only been adopted by a minority (only 4.3%) of the patients based on a survey which was carried out across 17 countries around the world [5].

For those patients who suffer from CAD, revascularization therapies including percutaneous coronary intervention (PCI) and coronary artery bypass surgery (CABG) have been the mainstay of treatment in addition to the use of lifelong cardiac medications including anticoagulants and beta-blockers [6]. Nevertheless, primary prevention has far been the best option.

Today, health services focus on maintaining people in their communities and therefore, nurse-led clinics which are similar to small outpatient clinics have been set up. Nurse-led clinics were first set up in the United States and United Kingdom in the year 1980 in the primary care setting in order to maintain patient care even after that they were discharged from the hospital following cardiovascular interventions [7]. These clinics were ruled by advanced nurse practitioners, clinical nurse specialists and other specialist nurses to provide efficient care to respective patients. Nurse-led clinics consist of specialized nurses who have been trained to counsel patients based on a primary treatment to prevent chronic diseases [8].

Nurses play a vital role in cardiovascular care by fostering and promoting healthy lifestyles and thus helping in reducing cardiovascular risks among the population [9]. Those clinics were independently run only by nursing staffs, and they were intended to mainly provide supportive, educational, preventive measures and psychological support to the patients, which were completely different from therapeutic clinics [10].

However, even though nurse led intervention showed major contributions in the efficacy of managing patients with cardiovascular diseases, only few studies have been published based on nurse-led clinics and cardiovascular diseases. Therefore, through this brief literature review, we aimed to show the implication of nurses and their interventions in the management of patients with cardiovascular diseases.

## Methods

### Literature sources and search strategies

Electronic databases including MEDLINE, EMBASE, Web of Science, Google scholar, and Cochrane database were searched for studies that were based on nurse-led interventions and cardiovascular diseases.

The following search terms or phrases were used:

- “nurse-led intervention and cardiovascular diseases”; “nurse-led intervention and coronary artery diseases”; “nurse-led intervention and percutaneous coronary intervention”; “nurse-led intervention and coronary artery bypass surgery”; “nurse-led intervention and catheterization”; “nurse-led intervention and revascularization”; “nurse-led clinic and primary prevention”; “nurse-led clinic and secondary prevention”; “nurse-led clinic and chronic conditions”.
- The word “nurse-led intervention” was also replaced by “nurse-led clinics” and “nurse” during this search process.
- During the search process, reference lists of relevant publications were also checked for suitable articles that could be included in this review.

### Criteria for inclusion and exclusion

Inclusion criteria involved any study published in English (randomized or observational or meta-analysis or systematic review or case study) that reported nurse-led interventions or nurse-led clinics that dealt with patients with cardiovascular disease whether as a primary prevention of the disease or in the treatment and management.

Exclusion criteria involved studies that did not involve nurse intervention with cardiovascular diseases, and studies that were irrelevant or duplicated studies. Publications related to nurse intervention and heart failure or atrial fibrillation or other cardiac conditions except cardiovascular diseases were excluded.

In a research based on comparison, there is an experimental group which is under study and there is a placebo which acts as the control group. In this literature review, several articles were included and the experimental group was the intervention group and included patients who were assigned to a nursing care, whereas the control group included patients who were assigned to the usual care without any nurse intervention.

### Compliance with ethical guidelines

This literature review is based on previously published studies and does not contain any study with human participants or animals performed by any of the authors.

All methods were carried out in accordance with relevant guidelines.

## Results

### Search outcomes

A total number of 845 publications were obtained. The title and abstract of relevant publications were carefully read and assessed. Irrelevant publications were directly eliminated (727). Studies involving nurse-led intervention in congestive heart failure (34) and atrial fibrillation (16) were eliminated. Duplicated studies were also eliminated prior to final selection of the studies (43).

Only publications based on the impact of nurse-led intervention or nurse led clinics on patients with cardiovascular diseases were selected for this review (25).

**Fig. 1:**
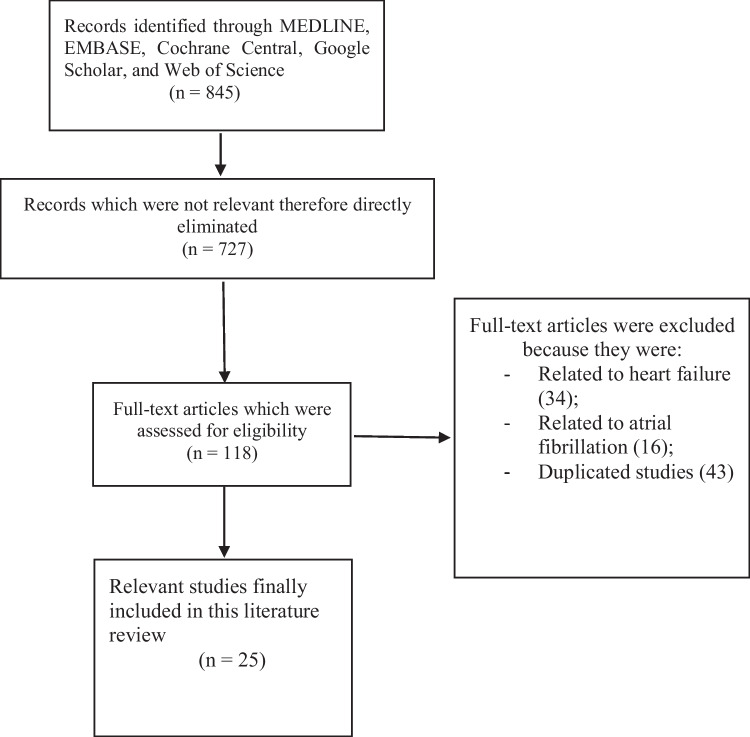
Represents the study selection based on the inclusion and exclusion criteria

Figure 1 represents the flow diagram for the study selection based on the inclusion and exclusion criteria.

Our current review was divided into several categories including nurse-led interventions in lifestyle program on cardiovascular risk factors, nurse-led interventions post coronary catheterization, nurse-led interventions in CABG and nurse-led interventions in other cardiac-related treatments and managements.

This is a descriptive literature review, therefore, any relevant publication can be discussed either in the introduction or in the discussion section of the paper as far as they have been cited.

Table 1 has summarized the studies [8, (11 – 29), 41, 43, 46, 47, 55] which have been described in this brief review.

**Table 1:** A summary of the studies which have been used in the description of this literature review

Studies	Study group with the No of participants (n)	Control group with the number of participants (n)	Type of study	Role of nurses
Page et al. [11]	49	49	Review	Lifestyle modification
McHugh et al. [12]	49	49	Randomized trial	Lifestyle modification
Su et al. [13]	73	73	Randomized trial	Lifestyle modification
Ruiz-Bustillo et al. [14]	39	39	Randomized trial	Lifestyle modification
Malek et al. [15]	86	78	Randomized trial	Lifestyle modification
Jjiang et al. [16]	52	50	Quasi-experimental design	Cardiac catheterization
Premkumar et al. [17]	31	31	Pilot randomized trial	Cardiac catheterization
Ibrahim et al. [18]	1325	-	Retrospective analysis	Cardiac catheterization
Adel et al. [19]	196	194	Randomized trial	Cardiac catheterization
Williams et al. [20]	100	-	Prospective observational cohort	Cardiac catheterization
Goodman et al. [21]	13	11	Observational study	Cardiac surgery
Kebapci et al. [22]	40	42	Quasi-experimental design	Cardiac surgery
Broers et al. [23]	27	26	Observational study	Cardiac surgery
Goodman et al. [24]	94	94	Randomized trial	Cardiac surgery
McLachlan et al. [25]	462	-	Retrospective study	Heart valve surgery
Cui et al. [26]	48	48	Randomized trial	Heart failure
Qiu et al. [27]	1571	1711	Meta-analysis	Congestive heart failure
Lee et al. [28]	66	25	Retrospective cohort	Heart transplant
Yan et al. [29]	116	119	Randomized trial	Atrial fibrillation
Laurant et al [8]	-	-	Systematic review and meta-analysis	Primary care
Mohan et al [30]	-	-	Pyramid model study	Tele-medicine
Zheng et al. [31]	86	87	Randomized trial	Lifestyle intervention
Buigues et al. [32]	282	344	Randomized trial	Secondary cardiovascular prevention
Al-Mallah [33]	4886	4954	Systematic review and meta-analysis	Clinical outcomes
carrington et al. [34]	-	-	Observational study	Primary prevention

## Discussion

### Factors contributing to the development of cardiovascular diseases

Several factors contribute to the development of CVD [35]. Cardiovascular risk factors include diabetes mellitus, hypertension, high body mass index (overweight and obese), cigarette smoking, a family history of cardiovascular diseases, dyslipidemia, lack of physical exercises, sedentary lifestyle including fast food intake and alcohol consumption. Those risk factors are more common in men. Menopause, and autoimmune diseases such as anti-phospholipid syndrome and systemic lupus erythematous might be additional risk factors in female patients [36, 37]. Age, and male gender were previously considered among major cardiovascular risk factors, however, nowadays it has been observed that even younger patients and also more and more women are at higher risk of cardiovascular diseases [38]. Patients with chronic kidney diseases are also at major risk of suffering cardiovascular episodes [39].

### Revascularization in patients with cardiovascular diseases

Patients with CAD might suffer from acute coronary syndrome (ACS) including STE myocardial infarction, NST elevated myocardial infarction and unstable angina [40]. Management of patients with ACS include invasive or surgical intervention [41]. PCI is the common invasive revascularization procedure carried out to treat patients with such conditions. In more complex diseases such as triple vessel disease, complex CAD, left main CAD and chronic total occlusion, open heart surgery (CABG in this case) should be considered [42]. Patients are then subject to lifelong antiplatelet agents or anticoagulants to prevent stent thrombosis and re-infarction [43].

### Nurse-led clinics

Health services are now focused on maintaining people in their communities and keeping them out of hospital wherever possible, making hospital settings available mainly for emergency patients [44]. Nurse-led clinics are small outpatient clinics which are monitored by experienced nurses in specific departments. Nurse led clinics have been shown to provide more efficient outpatient care and reduce waiting times [45]. These clinics are ruled by advanced nurse practitioners, clinical nurse specialists and other specialist nurses. These clinics have been set up since it was paramount to look for new ways to more effectively manage complex care to meet the requirements of patients and providers [34]. In addition, nurse-led clinics have been set up to increase patients’ access to care, to provide a cost-effective and high quality streamlined service [46]. Several nurse-led clinics have been set up for the management of patients with CVD [33].

### Nurse interventions in lifestyle programs on cardiovascular risk factors

In order to prevent or reduce cardiovascular risk factors, lifestyle modifications including healthy eating habits, sufficient daily physical activities, smoking cessation should be implemented [47]. Specialized nurses have been trained to counsel patients on the prevention and avoidance of cardiovascular risk factors [48]. The key roles of nurses are to educate patients on this lifestyle modification aspect. To support this statement, the EUROACTION Project which was a nurse-led model of cardiovascular prevention and rehabilitation programmes, and which was adapted in Spain has produced a healthier lifestyle. This model has achieved affordable and sustainable therapeutic goals in cardiovascular disease prevention in everyday clinical practice [32]. In another study based on the intervention of nurses, a significant number (31.5%) of patients with angina showed that they made changes to their diet, and a significant number of patients (23.3%) increased their daily physical exercises, more precisely walking [11]. Nurse-facilitated interventions were successfully shown to have reduced angina attacks, a number of physical disabilities and have reduced depression among the patients with CAD. In a trial conducted in Glasgow community practice clinics and in patients’ homes compared to the usual care for patients who were awaiting open heart surgery, there was a significantly higher rate of patients who stopped smoking with a rate reduction of 25% in the nurse-led group as compared to only 2% in the control group [12]. Cholesterol levels as well as high blood pressure were also significantly reduced in the nurse intervention group showing a favorable outcome. Moreover, a single-blinded randomized control trial based on the effects of a nurse-led eHealth cardiac rehabilitation program on health outcomes in 146 patients with CAD whereby nurses were implicated in lifestyle modification educational sessions with the patients during their hospital stay, and upon discharge, an e-platform helped those patients to better understand disease management, and monitor goal attainment for health behavioral changes [13]. This eHealth platform was monitored by nurses who then provided feedback about the patients on a weekly basis. It was demonstrated that at 6 weeks post-intervention, patients from the nurse-led interventions showed significant improvement in their daily physical activities, and health-promoting lifestyle profile as well as health-related quality of life compared to the control group favoring the application of eHealth nursing interventions to protect patients with CAD. Another randomized trial showed the benefit of a nurse-led follow-up among post discharge CAD patients for the achievement of good low density lipoprotein control [14]. The STEP-IT-UP study also showed nurse-led intervention to be an effective way to promote physical activities and improve cardiovascular risk factors within a 3-month period [15]. As such, it was an effective way physical activity could be promoted in sedentary elderly and the study demonstrated an improvement in cardiovascular risk factors within 3 months of implementation. Also, to note, in a two-armed randomized controlled trial, participants were randomly assigned to attend lifestyle intervention campaigns carried out by nurses versus receiving the usual care from hospital [31]. The participants were taught how to reduce the prevalence and adverse effects on cardiovascular health. The nurse-led Health Promotion Model guided lifestyle intervention program significantly improved the self-efficacy and implementation of health promoting behaviors among the participants.

### Nurse interventions post cardiac catheterization

Nurses play a key role in cardiac catheterization, especially post-operative. A quasi-experimental design study including 112 Chinese participants with myocardial infarction undergoing PCI, and based on a nurse-led individualized self-management program on health behaviors, control of cardiac risk factors, and health-related quality of life in such patients, demonstrated positive effects [16]. The study demonstrated that after a 1 year time period, significant improvements in health behaviors, quality of life, better control of cardiac risk factors including smoking cessation, decreased low density lipoproteins, and significant improvement in body mass index were observed among participants who were assigned to the nurse-led interventional group thus supporting nurse interventions in cardiac catheterization. In another study involving 62 participants who underwent cardiac catheterization, a nurse-led intervention group included discharge counseling and telephone follow-up, and the results showed that patients who were assigned to the nurse-led intervention group presented moderate to good smoking cessation, good adherence to cardiac medications, adherence to dietary changes, good physically active lifestyle and improved healthcare satisfaction [17]. In a retrospective analysis based on nurse-led clinics for 1325 patients with ST elevation myocardial infarction, non-ST elevated myocardial infarction, unstable and stable angina treated with PCI, 30-day mortality rate, re-admission and patients’ satisfaction were assessed [18]. The results showed that during the follow-up visit, mortality of 0.4% was recorded, and 10% of the patients were re-admitted within 30 days after follow-up, but among the 10% re-admission, only 1.8% was due to a cardiac cause. The study showed that nurse-led PCI clinics provided satisfactory management of cardiovascular risk factors without any increase in adverse cardiac outcomes. A randomized clinical study based on Interventional Cardiology Ward of the Jondi Shapur University Hospital in Ahvaz, Iran between years 2006 to 2008 on patients undergoing balloon angioplasty showed that nurse-led teaching significantly decreased complications following the invasive procedure [19]. Nursing staff teaching is simple, and this could significantly be effective in interventional cardiology to minimize complications after coronary interventions. In addition, the implication of nurse specialists in the assessment of radial artery prior to and after coronary catheterization using ultrasound in order to enhance planning and care has also shown positive response in Interventional Cardiology [20].

### Nurse-led interventions and cardiovascular surgery

The implication of nursing interventions in CABG has also shown to be effective. First of all, a randomized control trial consisting of 188 patients at a tertiary center in the United Kingdom evaluating a nurse-led support and educational program for patients who are waiting for CABG showed that the patients were very satisfied and appreciated support of the nursing staffs but communication among staffs and with the patients were suggested to be improved since patients’ appreciate physical and psychological preparations prior to surgery [21]. In a prospective study consisting of patients who were hospitalized for CABG between April 2014 and November 2015, in a hospital in Turkey, the authors demonstrated that the nurse-led clinical pathway was associated with an improvement in the length of hospital stay, that is, patients could be discharged earlier after the open heart surgery, and a significantly lower rate of complications was reported among the participants who were assigned to the nurse-led treatment group showing a benefit of nurse-led intervention following CABG [22]. Another study evaluated the safety and efficacy of a nurse-led clinic for patients who were recovering after CABG [23]. Five hundred eighty-four patients who underwent CABG were admitted to the hospital after the surgery. These patients were treated by either a nurse practitioner or a resident. Results of the study showed that those patients who were treated by the nurse practitioner were discharged from the hospital significantly sooner, without any increase in mortality rate among the patients. The authors concluded that a nurse-led clinic for patients who were recovering from CABG was safely and efficaciously introduced in a Dutch non-cardiac surgery hospital. Another study showed nurse-led interventional program to minimize overall healthcare utilization in patients who were awaiting cardiac surgery [24].

### Impact of gender on cardiovascular risk and the implication of nursing expertise to improve this condition

Cardiovascular prevention is applied differently between male and female patients and this has been underlined by various literatures. Men and women are not always treated the same way in clinical settings. The Lancet women and cardiovascular disease Commission aimed to improve cardiovascular prevention and reduce global cardiovascular mortality and complications in women by 2030 [49]. Cardiovascular mortality contributed to 35% of the total deaths of female patients in the year 2019 [50]. Throughout the literature, it has been shown that higher risk of bleeding in women than men was linked to these anticoagulation therapies. Cardiovascular diseases in women have often been understudied, underdiagnosed, under-recognized and undertreated all around the world. Treatment of cardiovascular diseases often include anti-platelet and anticoagulants. Women have often been underrepresented in or excluded from cardiovascular clinical trials and this has resulted in a reduction in the ability to measure safety and efficacy of therapies for women, the ability to identify sex-specific differences in vital outcomes, and the development of sex-specific strategies that could improve guideline recommendations for the prevention and management of cardiovascular diseases [51]. It has also been shown that women with diabetes mellitus were more likely to be assigned a lower cardiovascular risk category and to receive lifestyle counselling as well as less intense cardiovascular therapy when compared to men [52]. For a better quality of care in both men and women, nurses should also be involved in educating men and women about the specific potential risk factors which might be different in both genders. Another study where treatment-related sex differences has been described is a narrative review based on physical activity and diet in older women and was published in the Journal of Clinical Medicine [53]. Since there were different responses between exercise and diet between men and women, and the body’s response to exercise and to different nutrients as well as the choice of foods is different between the two sexes, the review showed a lack of studies based on women and the authors requested a need for more studies based on women and cardiovascular risk. The implication of nursing staffs to correctly identify cardiovascular risk factors in female patients might decrease the complication rate among similar patients.

### The impact of the recent pandemic (COVID-19) on the functioning of the health system and the implication of nurses to improve tele-medicine modalities

The recent COVID-19 pandemic has brought several changes to the functioning of the health system. The main aim was to maintain continuity of care for the patients. The health care industry was compelled to re-evaluate traditional methods of health care delivery. During this pandemic period, it was imperative to train nursing students to prepare them for evolving methods of care delivery, most specifically for telemedicine [54]. This transition towards a rise in the use of telemedicine modalities which was accelerated by the COVID-19 pandemic promises to be permanently applicable and represents and evolution in medical technology. Several other online applications which could act as primary prevention programs have facilitated management of patients. For example, technology-enabled diabetes prevention programs including physical activity, diet control, coaching, and so on were accepted and found to be useful by patients [55]. The acceptance of this new technology could be beneficial in so many ways, and could re-inforce clinical advice and sustain health behaviours advocated by nurses. Diabetes-trained nurses could contribute to the continual risk assessment, monitoring and timely intervention to prevent diabetes and potential complications indirectly preventing cardiovascular diseases.

During the COVID-19 pandemic, telemedicine was an effective method to maintain continuous care of the patients with cardiovascular diseases. A two-stage tele-consultation strategy with the implementation of a nurse-led tele-consultation strategy to manage over 12, 000 patients with cardiovascular diseases in Punjab, India, was set up [30]. Based on this tele-communication, patients were referred to physicians for uncontrolled diabetes and high blood pressure, as well as congestive heart failure. The study showed that this nurse-led tele-communication strategy was feasible to implement in restricted setting for triage of patients with cardiovascular diseases.

There is a huge pressure on the health care system due to chronic diseases. Competent health care providers including nurses can offer comprehensive care to patients during rehabilitation after being discharged from the hospital. In a recent systematic review and meta-analysis based on nurse-led telehealth intervention for rehabilitation (Tele-rehabilitation) among community dwelling patients with chronic diseases, the authors demonstrated that telephone follow-ups by nurses were the most commonly accessible tele-rehabilitation delivery approach which involved nurses-patient communications, self-management support, and regular follow-ups [56]. However, the authors stated that this nurse-led tele-rehabilitation program design would require upgrade.

### Other implications of nurse interventions in cardiology

A nurse practitioner clinic could offer a systematic approach to promote guideline adherence following heart valve surgery [25]. Another study showed a nurse-led educational program could significantly improve medication adherence, dietary modifications, social support and symptom control among Chinese patients with congestive heart failure [26]. Hospital re-admission was also significantly reduced. The authors concluded that the implementation of such nurse-led educational program could be of great value and be associated with better patients’ satisfaction and improved cardiovascular outcomes among patients with heart failure, especially among those patients who might not have regular access to cardiac hospitals and centers as per metropolitan population. Even in a recently published meta-analysis, the authors demonstrated that congestive heart failure patients who were assigned to a nurse-led intervention group had a significantly lower rate of re-hospitalization and mortality supporting its implementation [27]. In addition, a retrospective cohort study based on clinical outcomes of a nurse-led post discharge education program for 136 heart-transplantation patients showed a significant decline in outpatient visits with clinical problems, and a longer time interval until first unplanned re-hospitalization indicating an effective strategy of nurse-led educational programs after heart transplantation [28]. Even in patients with atrial fibrillation, nurse-led multidisciplinary team management has shown to reduce hospitalization due to a cardiac cause, and has significantly improved quality of life in patients with atrial fibrillation, suggesting that this innovative management approach should be implemented in clinical practice [29].

## Conclusions

Our review shows important implications of nurses in primary and secondary preventions of cardiovascular diseases, in cardiovascular interventions and in cardiac surgeries. They also have major roles during the management of cardiac complications including congestive heart failure, atrial fibrillation and heart transplantation. Nurse-led interventions are vital, and should be implemented in clinical practice for the treatment and management of patients with cardiovascular diseases. Based on advances in therapy, more research should be carried out to further investigate the effect of nurse-led clinics during the long-term treatment and management of patients with cardiovascular diseases.

## Data Availability

This is a literature review. No data was used for any statistical analysis. This is a literature review, without any data included. Therefore, there was no supplementary file for data associated with this article. References have been included in this published article when any study was cited.

## Abbreviations

NI: Nurse Interventions
CVD: Cardiovascular diseases
CAD: Coronary artery disease
CABG: Coronary artery bypass grafting
PCI: Percutaneous coronary intervention
